# Challenging Medical and Surgical Management of Ectopic Parathyroid Hormone (PTH) Adenomas in the Elderly

**DOI:** 10.7759/cureus.80230

**Published:** 2025-03-07

**Authors:** Anara Karaca, Koray Demirel, Bashir Mahamud, Furhana Hussein, Gideon Mlawa

**Affiliations:** 1 General Medicine and Diabetes and Endocrinology, King's College Hospital, London, GBR; 2 Nuclear Medicine, Ankara Teaching and Research Hospital, Ankara, TUR; 3 Internal Medicine and Diabetes and Endocrinology, Barking, Havering and Redbridge University Hospitals NHS Trust, London, GBR

**Keywords:** cinacalet, ectopic parathyroid adenoma, minimally invasive parathyroidectomy, parathyroid hormone washout, primary hyperparathyroidisim

## Abstract

Ectopic parathyroid adenomas (EPAs) are rare and difficult to localize, making surgical cures challenging on the first attempt. However, the management of EPAs in the literature on the geriatric population is limited. Herein, we report two elderly cases of EPAs in atypical locations and challenging management with a mini review of the literature.

An 82-year-old English male presented with confusion and constipation, and his tests confirmed primary hyperparathyroidism (PHPT). However, IV fluids and IV bisphosphonate treatment did not treat his hypercalcemia and imaging investigations could not localize parathyroid adenoma (PA); therefore, he underwent explorative left-sided parathyroidectomy, which confirmed normal parathyroid gland. His hypercalcemia improved shortly after surgery; nonetheless, a month later, he was readmitted with recurrent hypercalcemia symptoms. Single-photon emission computed tomography (SPECT) showed a nodule in the mediastinum referring to EPA. But the patient refused the second mediastinal surgery, and after MDT discussion, medical treatment with Cinacalcet was started. Finally, his calcium was normalized under this treatment and remains so.

A 69-year-old Turkish female presented with generalized bone pain, and she was diagnosed with PHPT. Her imaging investigations showed only one 1.6cm nodule in the thyroid, and no PA was detected. Further, fine needle aspiration parathyroid hormone (PTH) wash-out from that thyroid nodule showed very high PTH levels, referring to the thyroid's EPA. Meanwhile, she was screened for complications of PHPT, and nephrolitiasis and kyphosis due to severe osteoporosis were found. She underwent minimally invasive surgery under local anesthesia due to severe kyphosis for this EPA, which was confirmed by histopathology. Her PTH and calcium levels normalized shortly after surgery.

In limiting imaging results, PTH wash out could be helpful in diagnostic challenges, especially in suspected nodules/lesions in the neck. For the elderly, the value of surgery in achieving a cure is less straightforward than for the young. Thus, treatment of EPAs in the elderly should be decided based on comorbidities, difficult atypical localizations of EPAs and patients' preferences.

## Introduction

Ectopic parathyroid adenoma (EPA) is one of the rare causes of primary hyperparathyroidism (PHPT), which is defined by increased serum calcium and parathyroid hormone (PTH) [1]. Ectopic parathyroid glands result from aberrant migration during the early stages of development, and they constitute a common etiology of persistent or recurrent hypercalcemia [2]. The parathyroid glands and the thyroid and thymus migrate from the pharyngeal pouches to the lower neck during the seventh week of gestation. Ectopic parathyroid adenoma has been reported in 10%-16% of cases, ranging from the thymus (24%-38%) to the retro-esophageal space (22%-34%), thyroid gland (7%-18%), mediastinum (6%-20%), and carotid sheath (2%-9%) [3]. Complications of long‐standing PHPT are osteoporosis, increased risk of fractures and chronic kidney failure due to nephrolithiasis.

Parathyroid ultrasound (US), Technetium 99m methoxy isobutyl isonitrile (99mTc-MIBI), and single-photon emission computed tomography (SPECT) are images of choices to confirm and localize EPAs. PTH washout from suspected adenomas could also help diagnose. Sometimes, EPA cannot be detected by 99mTc-MIBI, and a possible cause is insufficient oxyphil cells in PTH adenomas, as these cells usually enhance to retain 99mTc-sestamibi [4]. SPECT can be an appropriate choice for localization in this type of EPA.

Parathyroidectomy is a curative treatment for parathyroid adenoma (PA) and EPAs. According to National Institute for Health and Care Excellence (NICE) guidelines, a patient should be referred for surgery if they have symptoms of hypercalcemia, end-organ damage such as osteoporosis, kidney stones, or fractures, creatinine clearance <60 cc/min; 24-h urine for calcium >400 mg/d (>10 mmol/d) and an adjusted serum calcium level of 2.85 mmol/L or above [5].

Effective surgery depends on accurately identifying and removing parathyroid glands. Due to a weakness in the localization technique, this was historically accomplished by bilateral neck exploration [6]. Today, minimally invasive parathyroidectomy (MIP) with localization of a single adenoma is emerging as the standard of care [7].

Elderly with EPAs likely to be more challenging to manage than younger adults. Persistent hypercalcemia in the elderly may lead to significant functional deficits that can affect their safety and quality of life compared with healthy age‐matched subjects, as the management in that age group is not always straightforward. Here, we report two cases of elderly patients with EPAs with challenges in localization and management and a mini review of the literature.

## Case presentation

First case

An 82-year-old male was admitted to the emergency department in the UK with confusion. He had no focal neurology or signs of sepsis. He had been experiencing constipation before admission and was on laxatives. PHPT was diagnosed with elevated calcium of 3.12 mmol/L (2.2-2.6), PTH 9.6 pmol/L (1.6-6.9), and phosphate of 0.79 mmol/L (0.8-1.5 mmol/L). Urinary calcium was 8.2 mmol/24hrs (2.5-7.5 mmol/24hrs). Renal function was normal, creatinine 84 mmol/L, urea 4.9 mmol/L. However, localization studies such as the parathyroid US and 99-Tc MIBI failed to show PTH adenoma.

Due to hypercalcemia symptoms, neurocognitive impairment and intestinal dysmotility, i.e., constipation, he was started on IV fluids and IV Pamidronate. His symptoms improved with fluids, but as the effect of pamidronate is about four to five weeks, the team decided to follow up his calcium levels as an outpatient.

Unfortunately, his calcium level was above 3 mmol/L a month later. Thus, he underwent explorative surgery for a left-sided parathyroidectomy. After surgery, his calcium level decreased to 2.3 mmol/L and his PTH level was 10 pmol/L; however, after a month, his calcium level increased above 3 mmol/L again, and his PTH level increased to 14 pmol/L, and he was readmitted.

**Figure 1:**
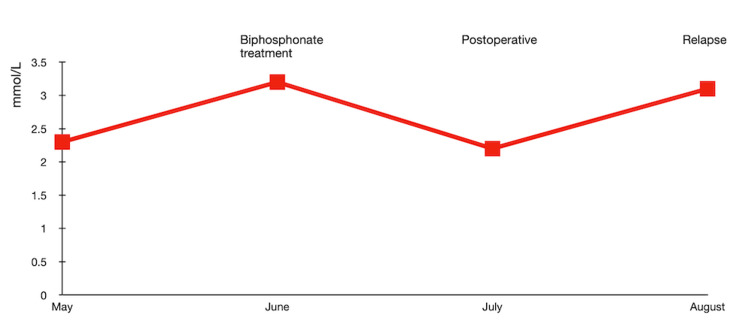
Serum calcium fluctuation during management.

Further investigations by chest SPECT showed an 11 mm nodule/node in the right subcarina. However, he refused a second surgery. Despite treatment with IV fluids and a few doses of intravenous pamidronate, his hypercalcemia persisted above 3 mmol/L (Figure 1).

In his last readmission, medical treatment with Cinacalcet 30 mg OD was commenced, which decreased his calcium levels to 2.34 mmol/L and PTH 5.9 pmol/L. He still remains normocalcemic under Cinacalcet.

Second case

**Figure 2:**
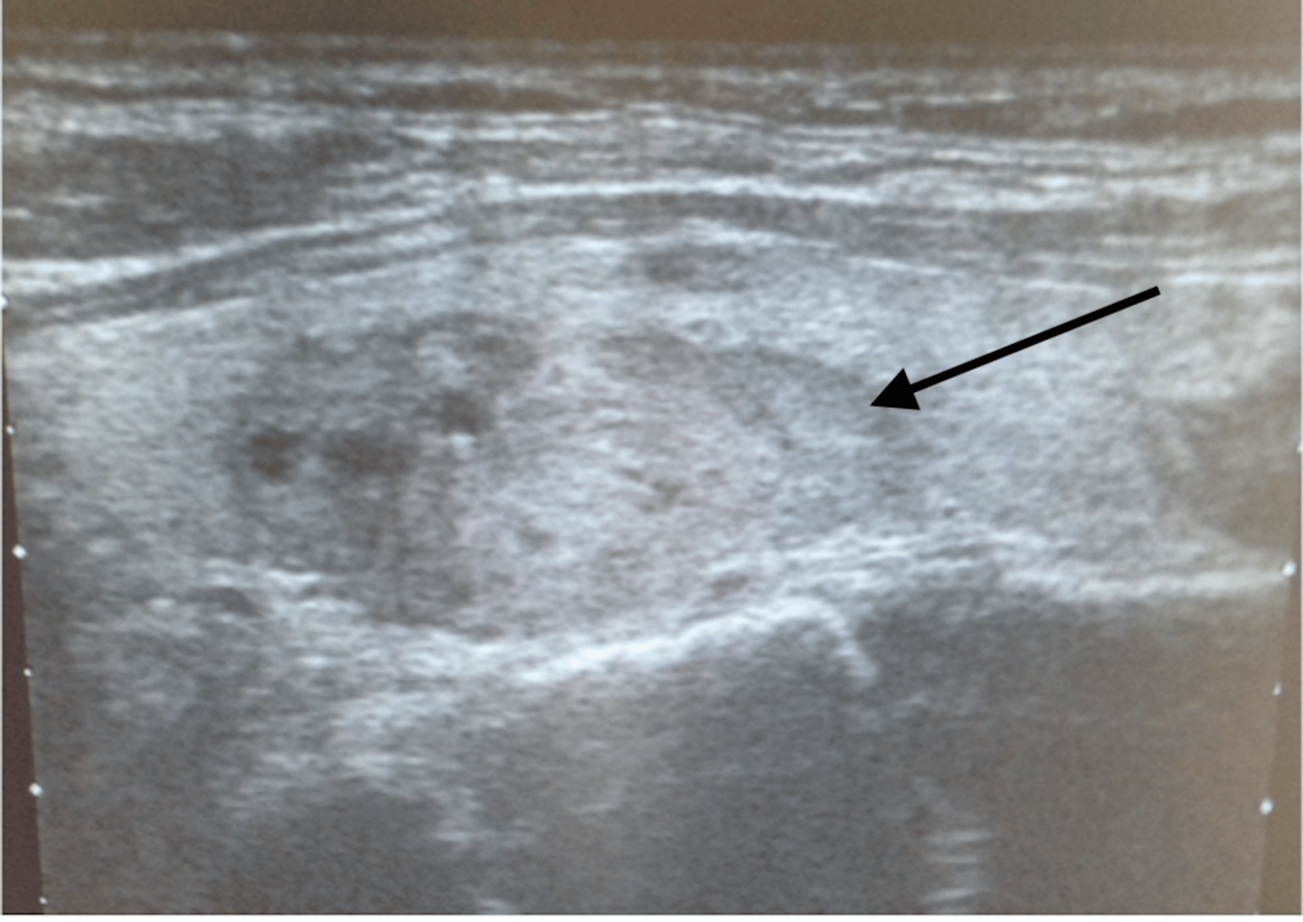
Neck US, 16x10cm hypoechoic nodule in left inferior thyroid lobe. Black arrow shows a hypoechoic nodule surrounded by thyroid gland. US: Ultrasound

A 69-year-old Turkish female was referred to the endocrine clinic in Turkiye with generalized bone pain. She was diagnosed with PHPT with elevated serum calcium (11.23 mg/dL (8.8-10.6)), reduced serum phosphate (1.82 mg/dL (2.5-4.5)), and her serum PTH was too high (1,581 pg/mL (14-72)), vitamin D deficiency (12.88 ng/mL (25-80)). She had a severe kyphosis due to osteoporosis, a lumbar T score of -3.8 and bilateral nephrolithiasis with generalized bone pain were long-term complications of PHPT. Pathological fracture was ruled out. A neck US showed four normal parathyroid glands and a 16x10cm hypoechoic nodule in the left thyroid gland (Figure 2).

**Figure 3:**
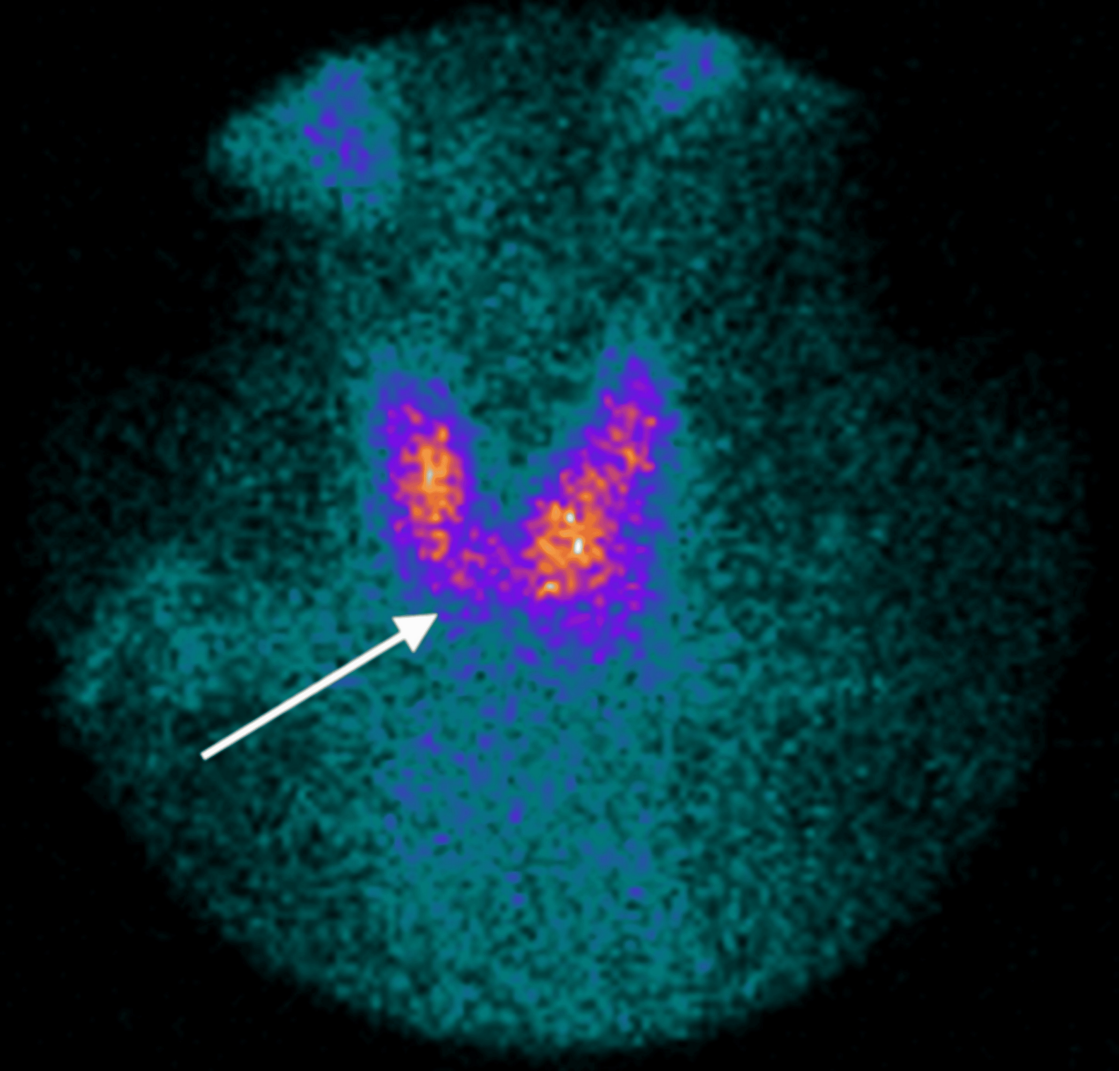
Thyroid iodine uptake with hypoactive nodule. The white arrow shows a hypoactive nodule in the left inferior thyroid lobe.

Thyroid iodine uptake showed a hypoactive nodule (Figure 3).

**Figure 4:**
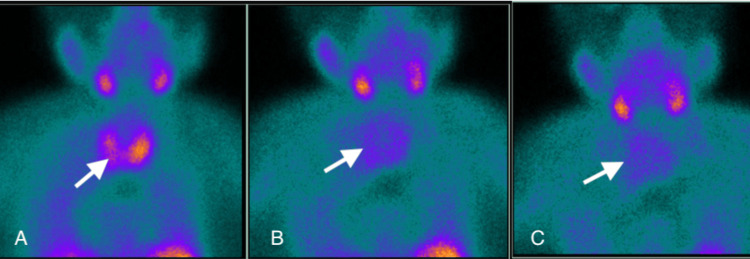
Negative Tc-99m MIBI scan for parathyroid adenoma. The white arrows show no uptake for parathyroid adenoma. (A) At 20 min, (B) at two hours, and (C) at four hours. MIBI: Methoxy isobutyl isonitrile

Interestingly, the dual Tc-99mMIBI scintigraphy was negative (Figures 4A-4C).

We did an FNA with PTH washout from the nodule in the left thyroid. Cytology showed inadequate cells, but the aspiration washout showed elevated PTH levels of >3,278 pg/mL (cutoff > 1,000 pg/mL).

**Figure 5:**
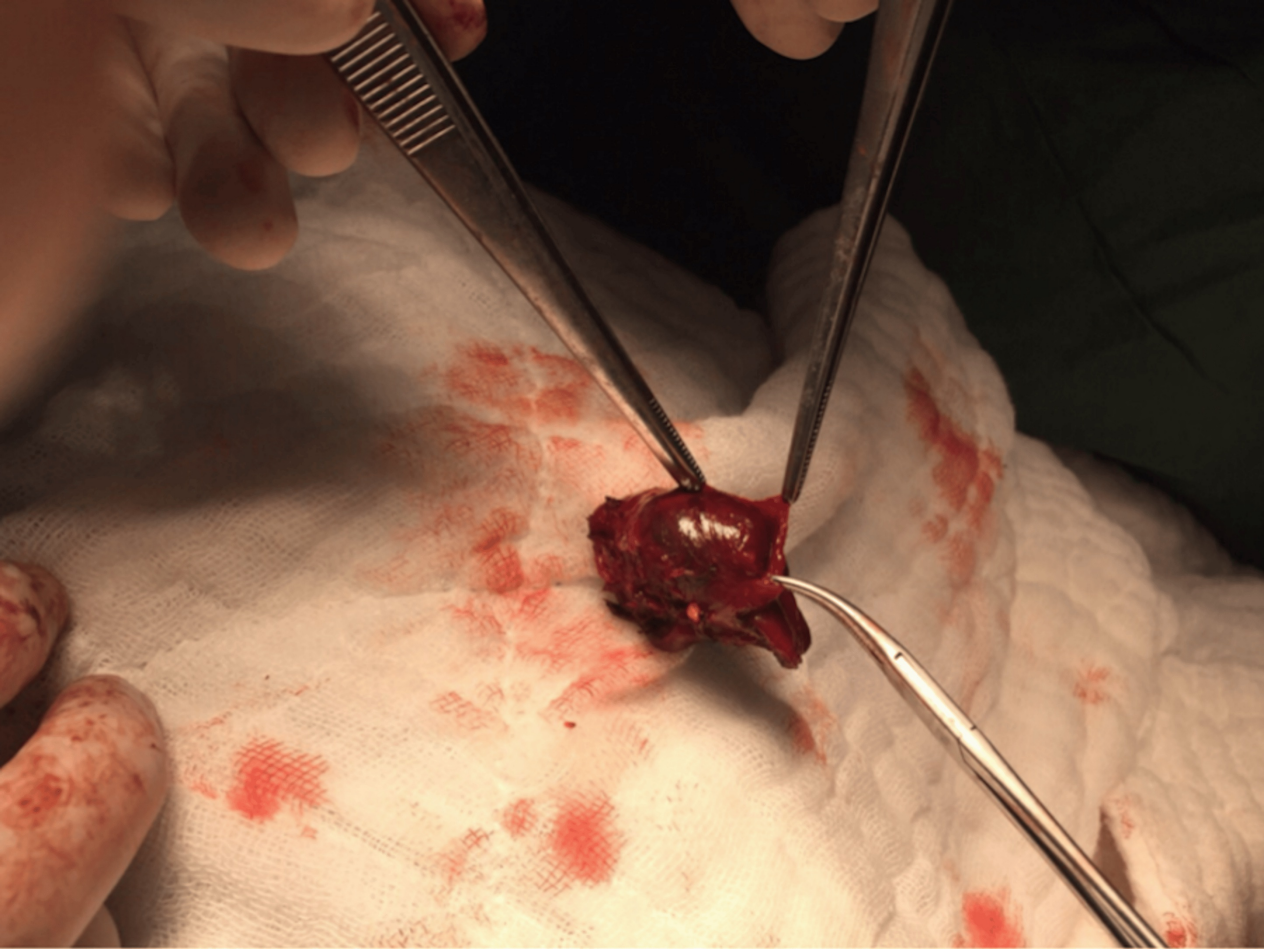
Left partial lobectomy of thyroid with PTH adenoma. PTH: parathyroid hormone

Hence, we localized ectopic PTH adenoma in the left lobe of the thyroid gland. Using a radio-guided probe for precise localization, she underwent a minimally invasive left thyroid partial lobectomy under local anesthesia due to severe kyphosis. She was awake during surgery, which lasted for 19 minutes. Left thyroid partial lobectomy is seen in Figure 5.

**Figure 6:**
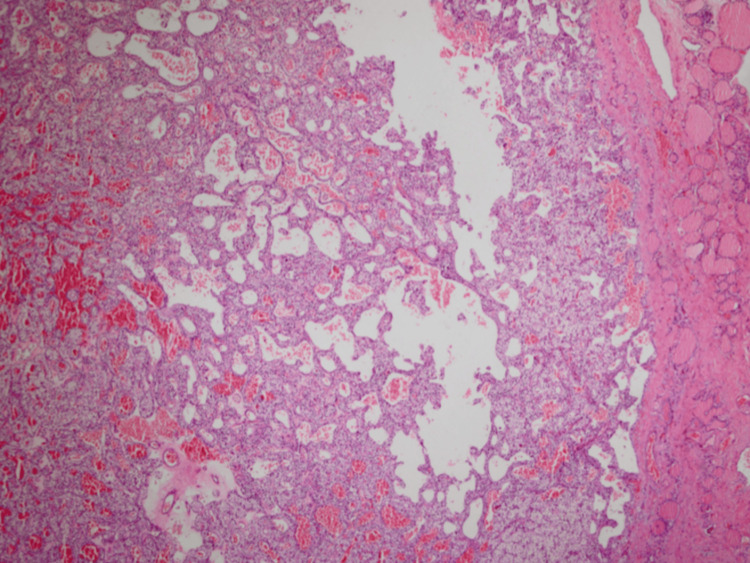
PTH adenoma. (H&E 100x and 400x); Histopathology PTH: Parathyroid hormone

Her histopathology confirmed a PTH adenoma surrounded by thyroid cells (Figure 6).

Twenty-four hours later, her serum calcium was 7.66 mg/dL (8.8-10.6), which was replaced by IV calcium. Her PTH decreased from 1,581 to 86.2 pg/dL (14-72 pg/dL). She continued with a short course of calcium supplementation, and bisphosphonate and vitamin D replacement treatments were added. Her calcium levels normalized after a week, and she remained stable for the next two years until she dropped from follow-up.

## Discussion

Parathyroid US, 99-Tc MIBI, and SPECT are the investigations that confirm and localize EPAs. However, some neck lesions are challenging to interpret, like our second case, which had inconclusive neck US and MIBI results. Further, fine needle aspiration (FNA) with PTH washout showed high PTH levels in that nodule, and later histopathology confirmed EPA. Thus, PTH washout can be highly reliable for differentiating EPA from other cervical lesions [8]. Physicians should consider PTH wash out in the limitations of imaging or false negative imaging results in the suspected EPA in the neck area, which can be a valuable diagnostic test. On the other hand, SPECT is a reasonable approach in mediastinal EPAs.

Surgery under general anesthesia is the preferred treatment option for EPAs. However, many face difficulties in the management of elderly patients with contraindications or refusal of surgery. Like our second patient, who had contraindications for general anesthesia due to her kyphosis, she underwent successful MIP with a gamma probe under local anesthesia. MIP under local anesthesia is rare but can be considered in selective patients. There were no other complications except transient hypocalcemia; she recovered well after surgery and was discharged the next day. MIP under probe can also be effective for mediastinal EPAs [9].

A gamma probe permits intraoperative localization of the diseased gland. It can only be used in a single PA. However, familial cases, known PTH carcinomas, previous neck radiation, and multiple adenomas are contraindications. The benefits of decreased complication risk, enhanced cure rates, and better cosmetic outcomes will likely continue to increase preference for MIP [7].

Surgery decisions for elderly patients should be made according to the location of the EPA, the patients' comorbidities, and the patients' preferences. Elderly with EPAs in difficult locations like in the mediastinum and comorbidities, which can be unfit for surgery, can go for medical treatment. Like our first patient, who refused the second mediastinal surgery, which made it difficult to manage his hypercalcemia. Bisphosphonates and IV fluids were not effective; thus, the patient was started on Cinacalcet 30 mg tablet daily, and his calcium was finally normalized.

Cinacalcet is not widely used, especially in patients unfit for surgery with EPA. It decreases calcium and PTH by increasing calcium sensitivity to PTH receptors. A recent meta-analysis by Chandran [10] confirmed that Cinacalcet was well tolerated. Moreover, it rapidly decreased calcium and PTH levels without increasing urinary calcium excretion, indicating its potential benefit as a medical treatment. Further, Minezaki et al. [11] showed that Cinacalcet reduced the size of PTH adenomas by 29% after six months of treatment. In another case report [12], it disappeared completely after 17 months of treatment.

Khan et al. [13] reviewed the rationale and evidence behind medical management and expectant monitoring. They found that medical management can be effective, and the patient's quality of life can be similar to those treated surgically. Under medical management, PTH and calcium can remain stable for 10 years.

The treatment doses of Cinacalcet used in the literature vary between 30 mg OD and 180 mg OD. However, physicians should be cautious about doses in elderly patients, as high doses may lead to symptomatic hypocalcemia requiring hospital admission [12]. Calcium levels should be monitored on long-term Cinacalcet treatment, and doses should be adjusted to prevent hypocalcemia. Our patient maintains normal calcium levels with Cinacalcet 30 mg daily.

## Conclusions

In diagnostic dilemmas of suspected nodules/lesions in the neck, whether they are EPA or not, PTH aspiration washout could be helpful. For the elderly, the value of surgery in achieving a cure is less straightforward than for the young. Thus, treatment of EPAs in the elderly should be tailored to each patient, including comorbidities, difficult localization of EPAs, and patients' preferences.

Our first case supports that medical management may be an acceptable option for the elderly age group with co-morbidities or refusal of surgery. In contrast, the second case highlights MIP as a possible option for managing EPA in the selected elderly groups.
